# Supplementary material for: Patient General Condition at Diagnosis: A Systematic Evaluation for Adults Diagnosed with Hematologic Malignancies
Source: J Pers Med. 2020 Aug 27;10(3):106. doi: 10.3390/jpm10030106 (PMC7564839; doi:10.3390/jpm10030106)
Supplement: Supplementary file 1 [file jpm-10-00106-s001.pdf]

APPENDIX

PATIENT GENERAL CONDITION AT DIAGNOSIS: A SYSTEMATIC EVALUATION FOR ADULTS DIAGNOSED WITH HEMATOLOGICAL MALIGNANCIES

Patient characteristics

The characteristics of the 27 patients excluded from the study were as follows:

- Gender: 15 male, 5 female
- Age distribution: range 50-91, median 75, 50-64 (6), 65-74 (4), 75 and over (12)
- Performance status (ECOG): PS-0 (5), PS-1 (6), PS-2 (4), PS-3 (5), PS-4 (0), not available (2)
- Diagnosis: acute leukemia (1), myeloproliferative neoplasms (0), myelodysplastic syndromes (3), chronic lymphocytic leukemia and related disorders (2), Hodgkin's lymphoma (1), other lymphomas 5, multiple myeloma(5), multicentric Castleman's disease (1), not available (2).

Research team characteristics

INVESTIGATOR	EXPERIENCE<br>IN HEMATOLOGY	EXPERIENCE<br>IN GERIATRICS	FAMILIARITY<br>WITH ASSESSMENTS
**Fernando Ramos**	Licensed Hematologist<br>Staff<br>Head of Clinical Hematology	Low	ECOG: High<br>LEE: High<br>GAH: Intermediate<br>CGA: Low
**Paola González-Carmona**	Resident (Hematology)	None	ECOG: High<br>LEE: Intermediate<br>GAH: Low<br>CGA: None
**María Isabel Porras-Guerra**	Low	Licensed Geriatrician<br>Staff	ECOG: Intermediate<br>LEE: Intermediate<br>GAH: Intermediate<br>CGA: High
**Sonia Jiménez-Mola**	Low	Licensed Geriatrician<br>Staff	ECOG: Intermediate<br>LEE: Intermediate<br>GAH: Intermediate<br>CGA: High
**Ana María Martínez-Peláez**	Licensed Nurse<br>(Hematology, Day Hospital)<br>Staff	Low	ECOG: Intermediate<br>LEE: Intermediate<br>GAH: Intermediate<br>CGA: None
**Agustín Blanco-Cabielles**	Licensed Nurse<br>(Hematology Ward)<br>Staff	Low	ECOG: Intermediate<br>LEE: Intermediate<br>GAH: Intermediate<br>CGA: None
**Saray Conde**	Licensed Nurse<br>(Hematology, Day Hospital)<br>Staff	Low	ECOG: Intermediate<br>LEE: Intermediate<br>GAH: Intermediate<br>CGA: None
**Abdolah Ahmadi**	Licensed Hematologist	None	ECOG: High<br>LEE: High<br>GAH: None<br>CGA: None
**Marta Castellanos**	Licensed Hematologist	None	ECOG: High<br>LEE: High<br>GAH: None<br>CGA: None
**Seila Cerdá**	Licensed Hematologist	None	ECOG: High<br>LEE: High<br>GAH: None<br>CGA: None
**Natalia de las Heras**	Licensed Hematologist<br>Staff	Low	ECOG: High<br>LEE: High<br>GAH: Low<br>CGA: None
**Elisa Menéndez**	Licensed Nurse<br>(Hematology Ward)<br>Staff	None	ECOG: Intermediate<br>LEE: Intermediate<br>GAH: Intermediate<br>CGA: None

INVESTIGATOR	EXPERIENCE IN HEMATOLOGY	EXPERIENCE IN GERIATRICS	FAMILIARITY WITH ASSESSMENTS
**Fernando Escalante**	Licensed Hematologist<br>Staff	Low	ECOG: High<br>LEE: High<br>GAH: Low<br>CGA: None
**Silvia Fernández-Ferrero**	Licensed Hematologist<br>Staff	Low	ECOG: High<br>LEE: High<br>GAH: Low<br>CGA: None
**Tamara Lado**	Licensed Hematologist	None	ECOG: High<br>LEE: High<br>GAH: None<br>CGA: None
**Violeta Martínez- Robles**	Licensed Hematologist	None	ECOG: High<br>LEE: High<br>GAH: None<br>CGA: None
**Filomeno Rondón**	Licensed Hematologist	None	ECOG: High<br>LEE: High<br>GAH: None<br>CGA: None
**Irene Padilla**	Resident (Hematology)	None	ECOG: High<br>LEE: High<br>GAH: None<br>CGA: None
**María Jesús Vidal**	Licensed Hematologist<br>Staff	Low	ECOG: High<br>LEE: High<br>GAH: Low<br>CGA: None
**María Lavinia Villalobos**	Licensed Hematologist	None	ECOG: High<br>LEE: High<br>GAH: Low<br>CGA: None
**Saad Yacoubi**	Resident (Hematology)	None	ECOG: High<br>LEE: High<br>GAH: None<br>CGA: None
**Francisco Javier Idoate-Gil**	Low	Licensed Geriatrician<br>Staff<br>Head of Department	ECOG: Intermediate<br>LEE: Intermediate<br>GAH: Intermediate<br>CGA: High
**José Antonio Rodríguez-García**	Licensed Hematologist<br>Staff<br>Head of Department	Low	ECOG: High<br>LEE: High<br>GAH: None<br>CGA: None

Supplemental Table 1. Research team members professional expertise and familiarity with tested assessments.

Results

ECOG score	n	%
**0**	57	31.3
**1**	79	43.4
**2**	26	14.3
**3**	15	8.2
**4**	5	2.7
**TOTAL:**	**182**	

Supplemental Table 2. Patient distribution according to ECOG Performance Status categories.

LEE score	n	%
0-5	77	47.5
6-9	49	30.2
10-13	30	26.5
14+	6	8.2
TOTAL	162	
	Range: 0-21, p25= 4, p50= 6, p75= 9, p90=11	

Supplemental Table 3. Patient distribution according to Lee Index (LEE) categories.

GAH score	n	%
0-42	54	49.5
43+	55	50.5
TOTAL	109	
	Range: 0-89, p25= 10·5, p50=43, p75= 67, p90=76	

Supplemental Table 4. Patient distribution according to Geriatric Assessment in Hematology (GAH) categories.

CGA categories	n	%
Robust	33	57.9
Vulnerable	19	33.3
Fragile	5	8.8
TOTAL	57	

Supplemental Table 5. Patient distribution according to Comprehensive Geriatric Assessment (CGA) categories.

Reasons	n	%
Patient not sent to Geriatrics	16	59.3
Patient non show at Geriatrics	5	18.5
Early death	6	22.2
TOTAL	27	

Supplemental Table 6. Reasons why patients over 75 did not underwent CGA.

Diagnosis	ECOG<br>Cutoff 3+	LEE<br>Cutoff 6+	GAH<br>Cutoff 43+	CGA<br>Not robust
	Patient proportion for each diagnostic subset			
Global	11.0	52.5	50.5	42.1
MPN	0.0	57.1	12.5	33.3
MDS	14.8	69.2	38.1	23.1
Acute leukemia	18.2	50.0	63.6	25.0
Hodgkin's lymphoma	10.0	42.3	0.0	72.2
Other lymphomas	16.7	100.0	55.6	100.0
CLL and related disorders	0.0	45.0	37.5	15.4
Multiple myeloma	18.5	57.7	76.5	50.0
p-value	0.13	0.27	<0.03	<0.01

Supplemental Table 7. Proportion of unfavorable features for the different scales according to diagnostic subsets.

ECOG, Performance status according to ECOG scale. LEE, Lee's Index for Older Adults. GAH, Geriatric Assessment in Hematology. CGA, Comprehensive geriatric assessment. MPN, Myeloproliferative neoplasms. MDS, Myelodysplastic syndromes (including MDS/MPN). CLL, Chronic lymphocytic leukemia.
